# Predicting Loss of Efficacy after Non-Medical Switching: Correlation between Circulating TNF-α Levels and SB4 in Etanercept to SB4 Switchers and Naïve Patients with Rheumatic Disease

**DOI:** 10.3390/jpm12071174

**Published:** 2022-07-19

**Authors:** Maurizio Benucci, Arianna Damiani, Edda Russo, Francesca Li Gobbi, Valentina Grossi, Amedeo Amedei, Maria Infantino, Mariangela Manfredi

**Affiliations:** 1 Rheumatology Unit, Hospital S. Giovanni di Dio, Azienda USL—Toscana Centro, 50143 Florence, Italy; ariannadam@outlook.it (A.D.); francesca.ligobbi@uslcentro.toscana.it (F.L.G.); 2 Department of Clinical and Experimental Medicine, University of Florence, 50134 Florence, Italy; edda.russo@unifi.it (E.R.); amedeo.amedei@unifi.it (A.A.); 3 Immunology and Allergology Laboratory, Hospital S. Giovanni di Dio, Azienda USL—Toscana Centro, 50143 Florence, Italy; valentina2.grossi@uslcentro.toscana.it (V.G.); maria2.infantino@uslcentro.toscana.it (M.I.); mariangela.manfredi@uslcentro.toscana.it (M.M.)

**Keywords:** non-medical switching, Etanercept/SB4, biosimilars, disease activity, rheumatic disease, precision medicine, loss of efficacy

## Abstract

Background: We investigated how the non-medical switching (NMS) between Etanercept (ETN)/originator and SB4/biosimilar affects treatment efficacy in a rheumatic disease (RD) cohort, evaluating some laboratory parameters as loss of efficacy predictors after NMS. Methods: We enrolled 124 patients with RD (rheumatoid arthritis, ankylosing spondylitis, psoriatic arthritis): 79 switchers from ETN/originator to SB4 and 45 naïve patients receiving SB4 (first biological treatment). At baseline, 6 (T1), and 12 months (T2), clinical and laboratory parameters were evaluated. Results: In naïve patients, TNF-α significantly increased at T1 in responders (NR) and non-responders (NNR). TNF-α was lower in NNR than in NR at T1 and T2. In NR and NNR, drug levels (DL) increased between T1 and T2. However, DLs were lower in NNR than in NR at T1 and T2. TNF-α was higher in switcher responders (SR) than in non-responders (SNR) at T1 and T2. In SNR, DLs were higher at baseline than in SR, but they decreased significantly at T1 and T2. Conclusions: We observed a decrease in DL and TNF-α levels after NMS in SNR. Moreover, in naïve patients, DL and TNF-α levels were higher in NR than in NNR. Monitoring DL and TNF-α levels may represent a future precision medicine approach to predict loss of efficacy after NMS.

## 1. Introduction

As defined by the European Medicine Agency (EMA) [1], a biosimilar is a biological medicine highly similar to another already approved biological medicine (the “reference medicine”) that should be comparable to the respective originator in terms of pharmacokinetic and pharmacodynamic properties, immunogenicity, efficacy, and safety, despite some non-meaningful differences in molecular composition. Additionally, the US Food and Drug Administration's (FDA) definition was less detailed, defining a biosimilar as a biologic product that is “highly similar to” an approved biologic product (the “reference”, “originator”, or “bio-originator” product) and that has “no clinically meaningful differences” in safety or effectiveness as compared to the reference product [2].

The evidence of the comparable efficacy and safety of currently licensed biosimilars to the originators was provided by head-to-head, non-inferiority, randomized controlled trials (RCT) of patients with rheumatoid arthritis (RA) naïve to biologics, with psoriasis (PsA), and with ankylosing spondylitis (AS) [3]. According to these results, by extrapolation, biosimilars were approved for all indications released to the reference product. After the RCT-blinded phase, patients randomized in the originator’s arm were switched to the respective biosimilar with no significant loss of efficacy and no safety alerts [4]. Biosimilars were designed to reduce the heavy economic burden caused by the high costs of originators, thus allowing for the treatment of a higher number of patients [5] and driving down the prices of the reference biologics to be competitive in the pharmaceutical market. Non-medical switching (NMS) occurs when a patient, whose current therapy is effective and well-tolerated, switches for economic or other non-medical reasons, such as from an originator TNF-α (tumor necrosis factor alpha) inhibitor to its biosimilar [6]. Concerns have been raised that switching patients from reference medicines (originators) to biosimilars, or other structurally related biologics, may lead to an increased immunogenicity and consequential safety problems, or even a loss of efficacy [7]. Recent evidence shows that NMS can, in any case, lead to an increase in costs over one year, and this may be attributed to failure due to loss of efficacy or adverse events [8,9].

Etanercept (ETN) is a recombinant human TNF receptor p75Fc fusion protein, licensed for RA, PsA, AS (including radiographic and non-radiographic spondylarthritis and ankylosing spondylitis forms), polyarticular juvenile idiopathic arthritis (JIA), and adult and pediatric psoriasis [10]. Recently, SB4 was developed as a biosimilar to the reference product ETN [11,12], with equivalent clinical efficacy demonstrated through phase III randomized clinical trials in RA patients [13]. When compared with non-switched groups of patients treated with SB4 or ETN, patients who switched from ETN to SB4 (ETN/SB4) were not significantly different in terms of efficacy and safety up to 100 weeks of follow-up [13]. Nevertheless, in the recent literature, it is described that the rate of loss of efficacy or adverse events after the switch between ETN/originator and its biosimilar SB4 ranges from 7 to 17% [14], but reports of up to 26% are described [9]. However, a recent evaluation of 17 RCTs and 74 real-life studies has shown that only two studies meet 5/7 criteria to design an NMS study [15].

An early evaluation of the loss of efficacy after NMS may guide the choice of treatment, determining the optimal treatment regimen for each patient, according to a precision medicine approach. This approach could (i) drive a change in therapeutic strategy for patients with a higher risk of failure (after switching as well as in naïve patients) (ii) and save costs related to the reduction in work ability, admissions to the hospital, and the need to re-switch to more expensive drugs. However, there is currently no evidence of biomarkers predicting the loss of efficacy after NMS between ETN and SB4 and the lack of efficacy of SB4 in naïve patients.

In this context, the present study aims to investigate for the first time how NMS between ETN/originator and SB4/biosimilar affects treatment efficacy in a multi-RD cohort. Moreover, we evaluated the possible role of laboratory parameters as predicting biomarkers of loss of efficacy after NMS and of lack of SB4 efficacy in naïve patients.

In a scenario of increasingly limited economic resources for health, as expected in the near future, this pilot study on NMS between ETN and SB4 could pave the way for the early monitoring of other NMS in different diseases.

## 2. Materials and Methods

### 2.1. Patients

We evaluated a total of 124 patients with RD followed at the Rheumatology Unit of San Giovanni di Dio Hospital, Florence (Italy). Out of them, 79 switcher patients received SB4/biosimilar as a replacement of ETN/originator, and 45 naïve patients received SB4 as a first biological line of treatment. Out of the 79 switcher patients, 27 had RA, 24 AS, and 28 PsA. Concerning the 45 naïve patients, 14 presented RA, 12 AS, and 19 PsA. The demographic characteristics of the switcher and naïve patients, matched for age and sex, are presented in Table 1.

All patients gave their written informed consent according to the prospective nature of the study as per the Declaration of Helsinki and Italian legislation (Authorization of the Privacy Guarantor No. 9, 12 December 2013). All patients also signed the consent to NMS as per Resolution No. 450 2017 of the Tuscany Region.

### 2.2. Laboratory Evaluation

At baseline, all patients were evaluated for the following blood chemistry parameters: erythrocyte sedimentation rate ESR (VES-Matic CUBE 200, Diesse Diagnostica Senese spa, Monteriggioni, SI, Italy), C-reactive protein CRP (AU 1800, Beckman Coulter Inc., Brea, CA, USA), interleukin 6 (IL-6) (Human IL-6 Instant Enzyme-linked Immunosorbent assay; eBioscience, Bender MedSystem GmbH, Vienna, Austria), TNFα (Human TNF-alpha Quantikine Immunoassay; R&D Systems Inc, Minneapolis, MN, USA), serum calprotectin Myeloid-Related Protein (MRP) (Calprest, Eurospital, Trieste, Italy).

### 2.3. Detection of Drug Levels

To study the immunogenicity, the assays for in vitro detection of drug levels (DL) of ETN and anti-drug antibodies (anti-ETN) were also performed before and after the switch to SB-4/biosimilar, using the Enzyme-Linked Immunosorbent Assay (ELISA) method (Lisa-Tracker Duo ETANERCEPT, Theradiag, France). These assessments were repeated 6 (T1) and 12 months (T2) after the start of SB4 administration.

### 2.4. Activity Disease Status Evaluation

All 124 RD patients were clinically evaluated from a complete physical examination of the osteoarticular system. In particular, the value of DAS28 (Disease Activity Score) was calculated in subjects with RA (remission < 2.6; low disease activity 2.6–3.2; moderate 3.2–5.1; high disease activity > 5.1), ASDAS (Ankylosing Spondylitis Disease Activity Score) in those with AS (inactive < 1.3; moderate < 2.1; high < 3.5; very high > 3.5), and DAPSA (Disease Activity Index for Psoriatic Arthritis) in those with PSA (remission < 4; low activity 4–14; moderate activity 14–28; high activity > 28).

### 2.5. Health Assessment Questionnaire

To assess the ability to carry out the common activities of daily life, the Health Assessment Questionnaire (HAQ) was also administered. These assessments were performed at baseline, T1 (six months), and T2 (12 months) after initiation of therapy in naïve and in switcher patients.

### 2.6. Statistical Analysis

The descriptive statistics were expressed by the mean, standard error mean (SEM), and standard deviation (SD). The significance of all statistical analyses was defined as *p* < 0.05.

To evaluate the variation in the parameters from the baseline and at 6 and 12 months in the responders and non-responders, naïve and switchers, the Kruskal–Wallis test ANOVA was performed for non-parametric data and the Bonferroni test for parametric data, considering significant changes if *p* < 0.05. Comparison of DL, TNF-a, IL-6, and MRP between responder and non-responder patients was achieved thanks to the unpaired t test for parametric data and the Mann–Whitney test for non-parametric data.

The strength of predictivity of biomarkers (DL-MRP-TNF-IL-6) vs. outcome parameters of quality of life and disease activity (HAQ, ESR, CRP) was evaluated by linear regression in naïve and switchers, separately.

## 3. Results

### 3.1. Patients’ Characteristics

Out of 79 patients undergoing ETN/SB4 NMS, 53 patients successfully completed the 12-months follow-up of the study (SR), while 26 patients (33% of the total) had to interrupt SB4 treatment prematurely (SNR). Among these therapeutic failures, we found 19 cases of loss of efficacy of the drug and 7 cases of adverse reactions, represented by 2 episodes of psoriasis, 2 of hypertension, 1 cancer, 1 infection in the lower respiratory tract, and 1 other condition. Out of 45 SB4 naïve patients, 33 patients after one year of SB4 therapy were responders (NR), and 12 patients were non-responders (NNR). Out of them, 10 reported a loss of efficacy of SB4, and 2 experienced adverse reactions (1 hypersensitivity and 1 other condition). Therefore, treatment interruptions among naïve patients occurred in 27% of cases.

### 3.2. Longitudinal Evaluation of Clinical and Laboratory Biomarkers, Disease Activity, and Quality of Life

We first performed a longitudinal comparison at baseline, six, and twelve months of follow-up.

Regarding the examination of each RD activity score, the variation during the follow-up of DAPSA, DAS28, and ASDAS, in different cohorts, is shown in Table 2.

Only one SNR patient had ADA (anti-drug antibodies) positive at 6 and 12 months of follow-up.

Moreover, Table 3 shows the changes in clinical and laboratory biomarkers, disease activity, and quality of life parameters, in SR and SNR, as well as NR and NNR, respectively.

We observed that in NR, HAQ and CRP decreased significantly from 1.37 ± 0.0, 7 to 0.54 ± 0.02 and from 1.51 ± 0.25 mg/dL to 0.49 ± 0.06 mg/dL (*p* = 0.0001) at six months; while in SR, HAQ decreased later (between 6 and 12 months) from 0.59 ± 0.03 to 0.51 ± 0.009 (*p* = 0.0038).

**Figure 1:**
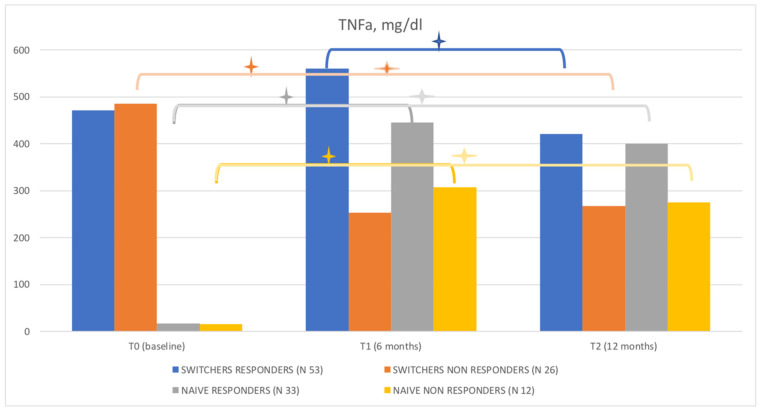
TNF-α levels in naïve patients and switcher responders and non-responders. The plus/star symbol (+) represent *p*-value = < 0.001.

In naïve patients, TNFα increased at T1 (*p* = 0.0001) from 16.66 ± 0.49 mg/dL to 445 ± 23.9 mg/dL in NR and from 15.28 ± 1.05 to 307.6 ± 59.18 mg/dL in NNR, without significant change at T2 (Figure 1). Finally, in NR, IL-6 decreased at T1 (*p* = 0.0001), without significant change at T2.

**Figure 2:**
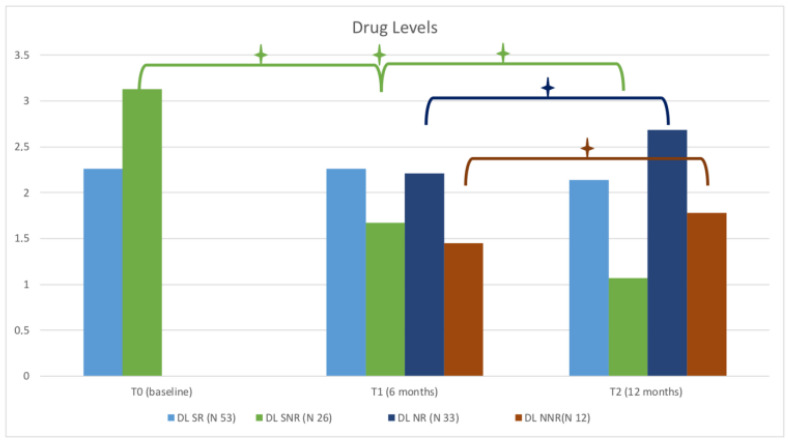
Levels of ETN in naïve patients and switcher responders and non-responders. The plus/star symbol (+) represent *p*-value = < 0.001.

Concerning DL (Figure 2), we observed an increase between T1 and T2 in NR, from 2.21 ± 0.14 mg/dL to 2.68 ± 0.2 mg/dL (*p* < 0.0032), and in NNR from 1.45 ± 0.41 mg/dL to 1.78 ± 0.17 (*p* = 0.024). Additionally, in SNR, DL decreased significantly at T1 from 3.13 ± 0.2 mg/dL to 1.67 ± 0.16 mg/dL (*p* < 0.0001). At T2, it further decreased to 1.07 ± 0.09 mg/dL (*p* = 0.02), while MRP increased between T1 and T2 (*p* = 0.002).

### 3.3. Comparison of Clinical and Laboratory Biomarkers, Disease Activity, and Quality of Life in ETN/SB4 Switcher Responders and Non-Responders and in Naïve Patients

We compared the above-mentioned biomarkers and parameters in the four cohorts of RD patients, notably, ETN/SB4 switchers and naïve SB4/SB4, in both the responder and non-responder conditions.

Regarding switcher patients, the main parameters that significantly differed in SR compared to SNR were:

- DLs were higher in SNR at baseline (3.13 ± 0.2 mg/dL vs. 2.26 ± 0.11 mg/dL *p* < 0.0001) but became lower than SR value at 6 months (T1) (1.67 ± 0.16 mg/dL vs. 2.26 ± 0.1 mg/dL, *p* = 0.069) and at 12 months (T2) (1.07 ± 0.09 vs. 2.14 0.1 mg/dL, *p* < 0.0001), due to the decrease in DLs at follow-up in this cohort.
- MRP in SNR was higher at T1 and at T2 (*p* < 0.0001).
- TNF-α was higher in SR than in SNR at T1 and T2 (559.6 ± 26.25 mg/dL vs. 253 ± 34.75 mg/dL, *p* < 0.0001, and 420.5 ± 27.62 mg/dL vs. 266.8 ± 24.67 mg/dL, *p* = 0.0002, respectively).
- IL-6 was higher in SR at T2 (*p* < 0.0001).

Regarding naïve patients, the main parameters that significantly differed between NR and NNR were:

- DL were lower in NNR at T1 (1.45 ± 0.41mg/dL vs. 2.21 ± 0.14, *p* < 0.0006) and T2 (1.78 ± 0.17 mg/dL ± 2.68 ± 0.2 mg/dL, *p* < 0.0047).
- MRP was higher in NNR at T2 (*p* < 0.018).
- TNFα was lower in NNR than in NR at T1 (307.6 ± 59.18 mg/dL vs. 445 ± 23.9 mg/dL vs. *p* = 0.032) and T2 (275.3 ± 49.17 mg/dL vs. 399.9 ± 24.5 mg/dL, *p* < 0.017).
- IL-6 was higher in NNR at T1 (*p* < 0.01) and T2 (*p* < 0.0046).

### 3.4. Predictability of the Biomarkers in Switcher and Naïve Patients

Finally, we analyzed the predictability (linear regression) of biomarkers (DL, TNFα, IL-6, and MRP levels) vs. the outcome parameters of quality of life and disease activity (HAQ, ESR, CRP) at baseline (T0) vs. T1 and T2 of follow-up in switchers and at T1 vs. T2 in naïve patients. Those correlations, for which it is necessary to reiterate the limitation imposed by the modest number of patients involved in this study, are directly proportional (positive predictability).

We observed that in switcher patients: IL6 correlated with HAQ at T1 in SR (*p* = 0.0028) and SNR (*p* = 0.01) patients; MRP correlated in SR patients with HAQ at T2 (*p* < 0.0001) and with CRP at T2 (*p* = 0.005) and, in SNR, with HAQ at T2 (*p* = 0.047).

Moreover, in naïve patients: MRP correlated with HAQ at T2 (*p* = 0.032) in NR, TNF-α with ESR (T2) (*p* = 0.032) and CRP (T2) (*p* = 0.021) in NNR, and IL-6 with ESR (T2) (*p* = 0.02) in NNR.

## 4. Discussion

The surprising data from this study demonstrated a difference between switchers and naïve patients at 6 months (T1) and 12 months (T2) of follow-up and showed the importance of new biomarkers in efficacy evaluation with the difference between the two populations examined. In responder patients, HAQ and CRP decreased at 6 months in the naïve cohort, while HAQ decreased later (between 6 and 12 months) in the switcher cohort. Another interesting fact is that in non-responder switcher patients, there is a progressive reduction in the circulating DL at 6 and 12 months. Only in naïve patients was TNF-α increased at 6 months in responders and non-responders and correlated with ESR and CRP in non-responder patients. Data in the literature on ETN drug levels are considered as low < 2.1 mg/L first quartile to high > 4.7 mg/L fourth quartile [16]. A correlation between circulating TNF-α levels and ETN clearance has recently been evaluated in healthy volunteers and in subjects with ankylosing spondylarthritis [17]. The interpretation of these data is not linked to the immunogenicity of SB4, as we have not observed the presence of anti-SB4 antibodies, neither in the naïve group nor in the switcher group. The data in the literature indicate that ETN can determine low immunogenicity between 4 and 6% in the switch studies; the different value obtained for the ETN originator (13%) compared to the biosimilar SB4 (0.8%) may be due to the electrochemiluminescence (ECL) method used [18,19,20]. The role of Methotrexate in reducing immunogenicity was observed for Adalimumab, with an improvement in the circulating DL [21]. Furthermore, the “Concerto” study shows that increasing doses of Methotrexate can lead to an improvement in disease activity in rheumatoid arthritis [22]. ETN only binds the trimeric form of TNFα forming an unstable 1:1 complex and can also bind a transmembrane TNF-α with a bond about twice as weak as the other anti-TNF. Furthermore, the absence of the IgG1 domain CH1 means that ETN does not have complement activation such as infliximab and adalimumab (CDC), whereas, due to the presence of the CH2 domain and CH3, it can exert weak antibody-dependent cytotoxicity (ACDC) by binding to the Fc receptor of NK cells. Therefore, the reduction in DL may correlate with this effect [23,24]. Glycosylation plays a significant role in the function, efficacy, clearance, and immunogenicity of a protein. ETN has 3 sites of N-glycosylation and 13 potential O-glycosylation sites. Two N-linked glycans, many of which are sialates, are found on the TNFR portion and an N-linked glycan in the Fc region. The O-linked glycan species also contains sialic acid. The glycan species in SB4 and the reference product were released enzymatically or chemically and chromatographically separated, quantified, and identified. The result suggested that the structure of each N-glycan species on SB4 was identical to that of the corresponding species on the reference product and that there was no specific N-glycan structure only for SB4 [24,25]. The structure and number of N-linked glycans in the TNFR region and in the Fc region differ. To compare the specific profiles of the N-linked glycans site, the glycopeptide profile obtained by peptide mapping was used. Three similar types of profiles have been derived for three glycosylation sites both in SB4 and in the reference product: Asn147 and Asn179 in the TNFR region and Asn317 in the Fc region [26]. The relative quantities of each species showed minimal differences between SB4 and the reference product. Since the mechanism of action of ETN is binding for TNF-α, only mannosylation was considered a critical quality attribute for the evaluation of glycosimilarity due to its influence on the serum half-life. Although significant quantitative differences were found in the sialate and core-fucosylated and galactosylated structures between the originator and the biosimilar, since the ADCC and CDC functions were not critical to the mechanisms of action of these products, this subset of the data was not considered in the evaluation of glycosimilarity. Mannosylation in humans, on the other hand, plays an important role in serum clearance, so the assessment of similarity based on the quantification of high mannose structures has represented an important critical quality attribute (CQA), which, based on our results, respected this particular biosimilar version of ETN (Benepali^®^) [27]. Serum calprotectin (Myeloid Related Protein, MRP) was higher in non-responder switcher and naïve patients and correlated with the poorest quality of life and, in switcher non-responders, increased after six months of treatment. A response to the MRP had already been demonstrated by the ETN originator [28]. A correlation between MRP and disability in patients with undifferentiated early arthritis, in particular with the anxiety-related component, has recently been demonstrated [29].

## 5. Conclusions

Currently, our data are the first in real life to indicate how, after NMS between ETN and SB4, a progressive fall in circulating DLs is observed in non-responder patients with a simultaneous decrease in circulating TNF-α levels. Moreover, the DLs and TNF-α levels are lower in non-responders than in responders, as well as in naïve patients. In this scenario, monitoring both DL and circulating TNF-α levels may represent a precision medicine approach to determine the loss of efficacy after NMS between ETN and SB4 and the lack of efficacy in naïve SB4 patients early. That could help in personalizing the RD therapy, speeding up treatment changes in non-responder patients, as well as preventing disease flare (avoiding NMS in patients with higher failure risk, switching back to ETN, or associating other drugs such as methotrexate or brief steroid treatments). On the other hand, high levels of TNF-α and high DLs can reassure both the clinician and the patient after NMS, as well as permit them to gradually reduce the frequency of follow-up visits, thus resulting in further economic savings for the National Health System. Our results in the evaluation of the loss of efficacy predictors in NMS could also be used to design future studies related to NMS in other diseases.

## Figures and Tables

**Table 1:** Demographic and clinical data of switchers and naïve patients (values expressed in mean ± DS).

	Number of Patients (N)	Age (aa)	Female/Male Ratio	Duration of Disease (aa)	Duration of Treatment (aa)
Switchers					
All	79	59.21 (±13.20)	45/34	9.11 (±3.75)	8.04 (±2.70)
RA	27	60.33 (±13.78)	20/7	9.44 (±3.64)	8.11 (±2.19)
AS	24	58.08 (±13.04)	6/18	9.62 (±4.27)	8.70 (±2.12)
PsA	28	59.11 (±13.15)	19/9	8.36 (±3.38)	7.43 (±2.8)
Naïve					
All	45	60.42 (±15.62)	32/13	3.33 (±2.56)	n.a.
RA	14	67.43 (±13.40)	12/2	4.79 (±3.15)	n.a.
AS	12	49.33 (±17.12)	8/ 4	2.42 (±0.67)	n.a.
PsA	19	62.26 (±12.8)	12/7	2.84 (±2.09)	n.a.

AS, ankylosing spondylitis; n.a., not applicable; PsA, psoriatic arthritis; RA, rheumatoid arthritis.

**Table 2:** DAPSA, DAS28, and ASDAS values of PsA, RA, AS patients (respectively) in switcher responder, switcher non-responder, naïve responder, and naïve non-responder cohorts.

Number of Patients (N)	Baseline (T0)	6 Months (T1)	12 Months (T2)
Switcher Responders (53)			
DAPSA (19)	2.20 (±1.79)	3.27 (±1.47)	3.84 (±0.94)
DAS28 (16)	2.23 (±1.18)	1.85 (±0.76)	2.04 (±0.40)
ASDAS (18)	2.71 (±1.21)	1.10 (±0.37)	1.41 (±0.20)
Switcher non-responders (26)			
DAPSA (10)	1.59 (±1.20)	5.20 (±0.42)	6.08 (±1.72)
DAS28 (10)	2.09 (±0.81)	3.25 (±0.91)	4.19 (±0.55)
ASDAS (6)	1.82 (±0.72)	2.52 (±0.42)	3.15 (±0.56)
Naïve responders (33)			
DAPSA (16)	9.91 (±7.60)	3.69 (±0.88)	3.97 (±0.13)
DAS28 (9)	4.33 (±0.99)	2.25 (±0.18)	2.98 (±0.60)
ASDAS (8)	6.36 (±4.76	1.30 (±0.00)	1.26 (±0.41)
Naïve non-responders (12)			
DAPSA (3)	11.1 (±7.16)	6.27 (±1.61)	8.43 (±0.51)
DAS28 (5)	5.09 (±1.61)	3.70 (±1.40)	4.15 (±1.39)
ASDAS (4)	3.65 (±0.24)	3.72 (±0.17)	4.47 (±0.39)

**Table 3:** Differences in clinical and laboratory biomarkers between baseline, 6 (T1), and 12 months (T2) of follow-up in switcher responder, switcher non-responder, naïve responder, and naïve non-responder patients (values expressed mean, SEM, and *p* significance < 0.05).

	T0 (Baseline)	T1 (6 Months)	T2 (12 Months)	*p*-Value T0–T1	*p*-Value T1–T2	*p*-Value T0–T2
Switcher responders (N 53)						
ERS, mm/h	22.47 (±1.8)	20.3 (±2)	18.36 (±1.66)	n.s.	n.s.	n.s.
CRP, mg/dL	0.47 (± 0.44)	0.61 (±0.1)	0.47 (±0.04)	n.s.	n.s.	n.s.
HAQ	0.53 (±0.01)	0.59 (±0.03)	0.51 (±0.009)	n.s.	0.0038	n.s.
MRP, ng/mL	2.25 (±0.09)	1.91 (±0.09)	2.09 (±0.1)	<0.0007	n.s.	n.s.
TNF-a, mg/dL	471 (± 26.18)	559.6 (±26.25)	420.5 (±27.62)	n.s.	0.0018	n.s.
IL 6, pg/mL	3.71 (±0.2)	3.9 (±0.3)	5.32 (±0.7)	n.s.	n.s.	n.s.
Drug levels mg/dL	2.26 (±0.1)	2.26 (±0.1)	2.14 (±0.1)	n.s.	n.s.	n.s.
Switcher non-responders (N 26)						
ERS, mm/h	19.96 (±2.1)	30.9 (±3.8)	29.88 (±3.28)	0.028	n.s.	n.s.
CRP, mg/dL	0.40 (±0.07)	1.10 (±0.18)	1.55 (±0.15)	0.001	n.s.	<0.0001
HAQ	1.58 (±0.9)	0.95 (±0.06)	1.41 (±0.05)	n.s.	n.s.	n.s.
MRP, ng/mL	2.18 (±0.14)	2.22 (±0.1)	2.88 (±0.19)	n.s.	0.002	0.002
TNFa, mg/dL	485 (±33.9)	253 (±34.75)	266.8 (±24.67)	<0.0001	n.s.	<0.0001
IL 6, pg/mL	3.28 (±0.18)	3.36 (±0.16)	4.96 (±0.79)	n.s.	n.s.	n.s.
Drug levels, mg/dL	3.13 (±0.2)	1.67 (±0.16)	1.07 (±0.09)	<0.0001	0.02	<0.0001
Naïve responders (N 33)						
ERS, mm/h	26.06 (±2.38)	19.36 (±2.1)	17.1 (±1.4)	n.s.	n.s.	0.01
CRP, mg/dL	1.51 (±0.25)	0.49 (±0.06)	0.46 (±0.05)	<0.0001	n.s.	<0.0001
HAQ	1.37 (±0.07)	0.54 (±0.02)	0.52 (±0.01)	<0.0001	n.s.	<0.0001
MRP, ng/mL	2.18 (±0.13)	2.12 (±0.1)	2.04 (±0.11)	n.s.	n.s.	n.s.
TNFa, mg/dL	16.66(±0.49)	445 (±23.9)	399.9 (±24.5)	<0.0001	n.s.	<0.0001
IL 6, pg/mL	5.64 (±0.59)	3.91 (±0.42)	4.03 (±0.51)	<0.0001	n.s.	<0.0001
Drug levels, mg/dL	n.e.	2.21 (±0.14)	2.68 (± 0.2)	n.e.	0.032	n.e.
Naïve non-responders (N 12)						
ESR, mm/h	33.42 (±5.72)	27.3 (±5.75)	29.75 (±4.09)	n.s.	n.s.	n.s.
CRP, mg/dL	2.21 (±0.87)	1.05 (±0.62)	0.85 (±0.25)	n.s.	n.s.	n.s.
HAQ	1.43 (±0.11)	1.18 (±0.09)	1.18 (±0.09)	n.s.	n.s.	n.s.
MRP, ng/mL	2.41 (±0.25)	2.15 (±0.1)	2.42 (±0.19)	n.s.	n.s.	n.s.
TNFa, mg/dL	15.28 (±1.05)	307.6 (±59.18)	275.3 (±49.17)	<0.0001	n.s.	<0.0001
IL 6, pg/mL	6.59 (±1.76)	5.63 (±1.86)	4.85 (±0.81)	n.s.	n.s.	n.s.
Drug levels, mg/dL	n.e.	1.45 (±0.41)	1.78 (±0.17)	n.e.	0.024	n.e

Legend: n.e., not evaluable; n.s., not significant.

## Data Availability

The data that support the findings of this study are available from the corresponding author, M.B., upon reasonable request.
