# Correction: Benucci et al. Predicting Loss of Efficacy after Non-Medical Switching: Correlation between Circulating TNF-α Levels and SB4 in Etanercept to SB4 Switchers and Naïve Patients with Rheumatic Disease. *J. Pers. Med.* 2022, *12*, 1174

**DOI:** 10.3390/jpm13020168

**Published:** 2023-01-18

**Authors:** Maurizio Benucci, Arianna Damiani, Francesca Bandinelli, Edda Russo, Francesca Li Gobbi, Valentina Grossi, Amedeo Amedei, Maria Infantino, Mariangela Manfredi

**Affiliations:** 1 Rheumatology Unit, Hospital S. Giovanni di Dio, Azienda USL-Toscana Centro, 50143 Florence, Italy; 2 Department of Clinical and Experimental Medicine, University of Florence, 50134 Florence, Italy; 3 Immunology and Allergology Laboratory, Hospital S. Giovanni di Dio, Azienda USL-Toscana Centro, 50143 Florence, Italy

The authors wish to make the following correction to this paper [1].

In the original article, one author Francesca Bandinelli was missing. The authors apologize for any inconvenience caused and state that the scientific conclusions are unaffected. The original article has been updated.
